# Three Cases of Umbilical Hemorrhage Occurring in the Same Family

**Published:** 1893-12

**Authors:** James Taylor


### THREE CASES OF UMBILICAL HEMORRHAGE OCCURRING IN THE SAME FAMILY.

BY

JAMES TAYLOR, M.R.C.S. Eng.

---

**Case I.**—1879. Sept. 29. Female, aged 13 days.

*History.*—She is the eighth child in an otherwise healthy family. Of the previous seven children six are living and healthy, the seventh died in infancy of whooping cough and pneumonia. There is no history of bleeding in parents or their children, but the mother's first cousin (father's brother's child) lost two of her children from umbilical hemorrhage.

This is a fine large baby. The cord was very thick and did not separate till seven days after birth. Before it separated, a visitor, in taking the child up to nurse it, seized hold of the front of the child's clothing, and since then the navel has been inclined to weep. The bleeding has set in more severely to-day (13th day), but there have been small clots coming away ever since the accident. The child is not at all blanched. There is slight venous oozing (enough to stain the bandage through after some hours). Directions were given that the bleeding surface should be dusted with tannic acid.

Sept. 30.—Bleeding continues, but is not more profuse. Tincture of matico applied in place of tannin. In the evening the hemorrhage became profuse. On consultation, strong perchloride of iron was applied with a pad and bandage, which stopped the bleeding for twelve hours.

Oct. 1.—Hemorrhage was now profuse. Solid perchloride of iron, ice, turpentine, pressure kept up manually, and the actual cautery were all tried with no effect, and the child died at 5 a.m. on October 2, three days after onset.

During the illness the child's elbows and ankles were noticed to become discoloured.

**Case II.**—1883. Dec. 19, Female, aged eight days. Labour was normal. The infant is a fine healthy-looking one.

This is the tenth child. The ninth had no trace of bleeding from the umbilicus, and lived to be fourteen months old, when it died of diarrhœa. The umbilical cord has not yet come off. The child was quite well at 5 a.m., when it was taken out of its cot, but at 8 o'clock the front of its night-dress was stained with blood. The child is blanched, and on stripping it all its clothes are found saturated with blood, and the remains of the cord are in the bandage. Above the umbilicus, for about an inch, the surface is red and the skin abraded; blood is oozing from this. Blood is also welling up from the bottom of the umbilical scar.

Pressure and styptics stopped the bleeding till the afternoon, when it recurred. Then, on consultation, two hare-lip pins were introduced under the umbilical scar, one from above downwards and the other transversely, and a thread passed round underneath. These effectually stopped the bleeding, and during the evening and next day the child rallied considerably; and with the exception of the tendency to bleed at the points of entrance and exit of the pins there was no bleeding.

However, during the evening of Dec. 22 (two days after the operation) the hemorrhage recurred, the blood welling up from the punctures, and in spite of tightening the ligature (which was successful for a time) and the application of styptics, the child died at 6 a.m. on Dec. 22, that is three days after the onset of the hemorrhage.

**Case III.**—1887. Nov. 10. Male, aged ten days. This is the twelfth child. The eleventh was stillborn, at full term; a breech presentation; death was caused by pressure on the cord.

Labour was perfectly natural. The cord came off on the seventh day.

This morning the nurse found a patch of dark-coloured blood on the rag over navel. On examination, the navel looks pretty healthy, but the edges are weeping a serous fluid. No blood is coming from it. With a view to euthanasia, and judging from past failures to stop the bleeding, the child was wrapped in a sheet of wadding, the nurse was to sit with it on her knee and whenever she saw a trace of bleeding to paint it with a solution composed of 1 oz. of tannin dissolved in 1 oz. of rectified spirit, and in the intervals of bleeding to keep a rag soaked in the solution on the navel.

Nov. 11. Child looks well. No bleeding beyond occasional oozing. Rag has hardened into an artificial scab over navel.

Nov. 12. Hardened rag remains in position. Nurse reports "a bruise on the child's shoulder this morning."

Nov. 13. When seen to-day there was free bleeding, but it was checked on removing the rag and applying the solution with a brush.

Nov. 14. Nurse says "the tannin solution restrains the hemorrhage much more effectually than either hazeline or Ruspini's styptic," with which she is supplied as alternatives.

Nov. 15. Bruise on shoulder is as big as a 5/- piece, and dark coloured.

Nov. 18. No bleeding for three days.

1888. Feb. 14. The child has had no trace of bleeding since, and is now passing through an attack of whooping-cough.

At time of writing the child is six years old, and is now a fine sturdy boy. He was not vaccinated till he was two years old, and then there was no more bleeding from the scratches than normal.

This disease appears to be more common in male children than female, but in these cases two were female and one male. The tendency to hemorrhage is transmitted through the female members of the same family, and this holds good here, for the only other case of hemorrhage in the family, as far as can be ascertained, is that the first cousin of the mother on her father's side lost two children from umbilical hemorrhage, *i.e.* the grand-fathers of the babies were brothers. It is curious that it is not till the eighth child in the family that the disease showed itself, and also that the intermediate child in the series of cases should show no sign of tendency to bleed; also that none of the other children have ever shown the slightest symptom of hæmophilia. The mother passed through her confinements without having any *post-partum* hemorrhage, and although at the menopause she suffered considerably from menorrhagia, she is now quite well.

Jaundice was not a marked symptom in these cases, though bruising is noted in the first and third cases.

The treatment with the solution of tannin in the last case was eminently satisfactory, and the tannin seemed to have more controlling influence over the hemorrhage than either of the other styptics that were tried; and perhaps the styptic renewed constantly as it was in this case had a better chance of acting directly on the bleeding point itself than when combined with pressure, as this could not be rendered effective owing to the abdominal walls being in constant movement.

It would be worth while in such cases to give chloride of calcium by the mouth, as suggested by Dr. Wright,[1] who describes chloride of calcium combined with fibrin ferment as the "physiological styptic." This treatment would seem specially desirable, as in an analysis of fœtal blood at the time of birth, given in the *Annual of the Universal Medical Sciences* for 1889,[2] it is stated that the blood is rich in salts, there being a marked preponderance of chlorides in the serum, so that an absence of salts in the blood might account for the occurrence of hemorrhage.

[1] *Brit. Med. Jour.*, Dec. 19, 1891. [2] Vol. ii., J-5.

